# Transcriptome analysis of immature xylem in the Chinese fir at different developmental phases

**DOI:** 10.7717/peerj.2097

**Published:** 2016-06-07

**Authors:** Yunxing Zhang, Xiaojiao Han, Jian Sang, Xuelian He, Mingying Liu, Guirong Qiao, Renying Zhuo, Guiping He, Jianjun Hu

**Affiliations:** 1 State Key Laboratory of Tree Genetics and Breeding, Chinese Academy of Forestry, Beijing, China; 2 Key Laboratory of Tree Breeding of Zhejiang Province, The Research Institute of Subtropical of Forestry, Chinese Academy of Forestry, Hangzhou, Zhejiang, China; 3 Institute of Architectural and Artistic Design, Henan Polytechnic University, Jiaozuo, Henan, China; 4 Key Laboratory of Tree Breeding and Cultivation of State Forestry Administration, Research Institute of Forestry, Chinese Academy of Forestry, Beijing, China

**Keywords:** Transcriptome, Chinese fir, RNA-Seq, Wood formation, Xylem

## Abstract

**Background.**Chinese fir [*Cunninghamia lanceolata* (Lamb.) Hook.] is one of the most important native tree species for timber production in southern China. An understanding of overall fast growing stage, stem growth stage and senescence stage cambium transcriptome variation is lacking. We used transcriptome sequencing to identify the repertoire of genes expressed during development of xylem tissue in Chinese fir, aiming to delineate the molecular mechanisms of wood formation.

** Results.** We carried out transcriptome sequencing at three different cultivation ages (7Y, 15Y and 21Y) generating 68.71 million reads (13.88 Gbp). A total of 140,486 unigenes with a mean size of 568.64 base pairs (bp) were obtained via *de novo* assembly. Of these, 27,427 unigenes (19.52%) were further annotated by comparison to public protein databases. A total of 5,331 (3.79%) unigenes were mapped into 118 pathways by searching against the Kyoto Encyclopedia of Genes and Genomes Pathway database (KEGG). Differentially expressed genes (DEG) analysis identified 3, 16 and 5,899 DEGs from the comparison of 7Y vs. 15Y, 7Y vs. 21Y and 15Y vs. 21Y, respectively, in the immature xylem tissues, including 2,638 significantly up-regulated and 3,280 significantly down-regulated genes. Besides, five NAC transcription factors, 190 MYB transcription factors, and 34 WRKY transcription factors were identified respectively from Chinese fir transcriptome.

**Conclusion.** Our results revealed the active transcriptional pathways and identified the DEGs at different cultivation phases of Chinese fir wood formation. This transcriptome dataset will aid in understanding and carrying out future studies on the molecular basis of Chinese fir wood formation and contribute to future artificial production and applications.

## Introduction

Chinese fir [*Cunninghamia lanceolata* (Lamb.) Hook.], a fast growing evergreen coniferous tree (2*n* = 2*x* = 22), is one of the most important native tree species for timber production in southern China and is also distributed in Vietnam. It is the third most commonly planted tree species in plantations worldwide (Del Lungo, Ball & Carle, 2006). Due to its high value in terms of adaptability, growth rate, timber quality, versatility and commercial value, the planting area of Chinese fir in China is around 9.215 million ha, accounting 28.54% of all forested land (Lei, 2005; Shi, Zhen & Zheng, 2010; Huang et al., 2012) and for 20–30% of the total commercial timber production in China (Li & Ritchie, 1999; Orwa et al., 2013). Chinese fir growth and development can be divided into three phases including fast growing stage, stem growth stage and senescence stage (Duan et al., 2004).

Wood formation involves various division and differentiation activities of cambium cells, including vascular cambium activation, secondary xylem differentiation, cell expansion, secondary wall deposition, programmed cell death, and heartwood formation (Ye & Zhong, 2015). Significant progress has been made in the past decade in uncovering the molecular players involved in the developmental phases of wood formation in tree species. *Populus trichocarpa* is the first sequencing tree (Tyler, 2006), creates opportunities for investigation of secondary growth, and secondary xylem (wood) development in woody plants (Brunner, Busov & Strauss, 2004; Cronk, 2005; Jansson & Douglas, 2007). Transcriptome analyses revealed that the suite of genes, highly expressed in wood-forming cells, includes receptor kinase, transcription factors, and secondary wall biosynthesis genes.

In transcriptional network, secondary wall NAC, MYB and WRKY transcription factors act as the top-level and second-level master switches, respectively (Zhong & Ye, 2014). These findings represent an important step toward elucidating the molecular mechanisms controlling wood formation. To date, genome sequences have been released for four tree species (Ye & Zhong, 2015), including the angiosperms *P. trichocarpa* and *Eucalyptus grandis* (Myburg et al., 2014), and the gymnosperms *Picea abies* (Nystedt et al., 2013) and *P. glauca* (Birol et al., 2013). Furthermore, transcriptome sequences have been obtained, for *Acacia auriculiformis* (Wong, Cannon & Wickneswari, 2011), *E. camaldulensis* (Thumma, Sharma & Southerton, 2012), *Fraxinus spp.* (Bai et al., 2011), *C. lanceolat*a (Huang et al., 2012), *Larix leptolepis* (Zhang et al., 2012), *Populus simonii × Populus nigra via* (Chen et al., 2012), *P. spp.* (Raherison et al., 2012), *Pinus monticola* (Liu, Sturrock & Benton, 2013), *P. glauca* (Raherison et al., 2015). The availability of these genome and transcriptome sequences together with an improvement of the methodologies used for generation of transgenic trees will enable researchers to directly employ tree species as models for studying wood formation.

Studies of Chinese fir have mainly focused on anatomical, biochemical, cytological, physiology, and ecological aspects, with only few reports relating to molecular mechanism of wood formation. Due to the limited genomic sources of Chinese fir, it is important to explore transcriptome for further molecular improvement. RNA sequencing (RNA-seq) provide a revolutionary tool with numerous applications for high-throughput functional genomics research (Mardis, 2008; Schuster, 2008; Morozova, Hirst & Marra, 2009; Nagalakshmi, Waern & Snyder, 2010). It has accelerated the investigation of the complexity of gene transcription patterns, functional analyses and gene regulation networks in plants (Wang et al., 2010b).

Transcriptome analyses (Huang et al., 2012; Qiu et al., 2013; Wang et al., 2013a) have identified a number of candidate genes and transcription factors correlated with changes in wood formation in Chinese fir. In the previous studies, however, the samples were collected from the same year or during the same cultivation phase.

In the present work, we used RNA sequencing (RNA-seq) technology to characterize the transcriptome at different stages of Chinese fir growth and development. The RNA samples from three different growth and development phases were sequenced with the high-throughput Illumina deep sequencing technique. Based on the bioinformatics analysis of assembled transcriptome data, we characterized immature xylem transcriptional pathways during the different cultivation phases of Chinese fir. Furthermore, we identified the DEGs subject to regulation during xylem development. The transcriptome sequencing of Chinese fir immature xylem may help to discover new genes and pathways. The data will promote future genetic and genomics studies on the molecular mechanisms of wood formation, and contribute to future applications, including artificial wood production.

## Materials and Methods

### Plant materials

The samples of Chinese fir [*Cunninghamia lanceolata* (Lamb.) Hook.] were collected from three different sites in Kaihua Country Forest Farm (29°08′33.56N, 118°23′56.59E), Zhejiang Province. No specific permits were required from the Forest Farm to select samples. The Forest Farm is not privately-owned and the field studies did not involve protected species. The immature xylem tissues were collected from three trees at every three different cultivation phases (7 years, 15 years and 21 years of cultivation (7Y, 15Y and 21Y)). The each phase, samples (outer glutinous 1–1.2 mm layer comprising early developing xylem tissue) were harvested from approximately breast height (1.0–1.20 m) on the main stem after removal of the bark using razor blades as described by Huang and Eshchar (Mizrachi et al., 2010; Huang et al., 2012). All of the tissue samples were immediately frozen in liquid nitrogen and stored at −80 °C for future use.

### RNA extraction, library construction and RNA-seq

Experimental procedures including sample preparation and sequencing were performed following the standard protocols (Illumina, Inc.). Total RNA was extracted separately from each sample using the R6827-01 Plant RNA Kit (Guduo, Shanghai, China). Three biological replicates were performed at three different cultivation phases. The concentration of RNA was analyzed using a spectrophotometer (UV-Vis Spectrophotometer, Quawell Q5000; Quawell, San Jose, CA, USA), and the integrity of RNA was evaluated with an Agilent 2100 Bioanalyzer (Agilent Technologies, Santa Clara, CA, USA). Equal quantities of high-quality RNA from each sample were combined into a single large pool for cDNA synthesis.

The mRNA-seq library was constructed using Illumina’s TruSeq RNA Sample Preparation Kit (Illumina Inc, San Diego, CA, USA). The mRNA isolation, fragment interruption, cDNA synthesis, adapter ligation, PCR amplification and RNASeq were performed at Beijing BioMarker Technologies (Beijing, China). The poly-A mRNA was enriched using oligo (dT) magnetic beads, and the mRNA was broken into fragments by fragmentation buffer. The cleaved RNA fragments were transcribed into first-strand cDNA using random hexamer primers, followed by second strand cDNA synthesis using DNA polymerase I and RNase H. The short fragments were purified with the QiaQuick PCR Purification Kit (Qiagen) and eluted in EB buffer for end-repaired by addition of poly(A) to 3^′^. Then the suitable fragments were separated by an agarose gel electrophoresis and selected for PCR amplification as sequencing templates. The constructed mRNA-seq library was sequenced on the Illumina HiSeq™ 2500 sequencing platform.

### Sequence data analysis and assembly

To obtain high-quality clean data for *de novo* assembly, the raw reads were filtered by removing the adapter sequences, low quality sequences (reads with ambiguous bases ‘N’), and reads in which more than 20% of bases had a *Q*-value <30. Reads were assembled using the reference transcriptome sequence of Chinese fir using the Bowtie and RSEM packages (Grabherr et al., 2011). The clean reads were assembled into contigs using Trinity ( http://trinityrnaseq.sourceforge.net/) (Grabherr et al., 2011). After Trinity *de novo* assembly and correction, the contigs without any gaps were linked into transcripts according to the paired-end information of the sequences. Related contigs were clustered into transcripts based on nucleotide sequence identity. The longest transcripts were regarded as unigenes redundancies were removed. Finally, the unigenes were combined to produce the final assembly used for annotation. The unigenes expression abundance was represented in reads per kilobase of exon model per million mapped reads (RPKM). The RPKM measure of read density reflects the molar concentration of a transcript for RNA length and for the total read number in the measurement.

### Functional annotation

To determine the functional annotation of the unigenes, the assembled sequences were compared against the NCBI Nr database (Deng et al., 2006), SwissProt (Apweiler et al., 2004), GO (Ashburner et al., 2000), COG (Tatusov et al., 2000), and KEGG (Kanehisa et al., 2004) with an *E*-value ≤10^−5^. Gene names were assigned based on the best BLAST hit (Altschul et al., 1997). Open reading frames (ORFs) were predicted using the “GetORF” program ( http://emboss.sourceforge.net/apps/cvs/emboss/apps/getorf.html). The longest ORF extracted from each unigene was defined as coding sequence (CDS), and the CDSs were translated into amino sequences using the standard codon table. The Blast2GO program was applied to obtain GO annotation of unigenes with an *E*-value ≤10^−5^ including molecular function, biological process, and cellular component categories. The unigenes sequences were aligned to COG database to classify and predict possible functions. Annotations of Chinese fir unigenes were used to predict biochemical pathways using the pathways tools. The KEGG database was used to analyze gene products related to metabolism and gene function in cellular processes.

### Detection of candidate SSR markers

The assembled sequences longer than 1 kb were used for the detection of SSR markers. Potential SSR markers were detected among the 17,902 unigenes using MISA software ( http://pgrc.ipk-gatersleben.de/misa/). The parameters were set for the identification of perfect dinucleotide motifs with a minimum of six repeats, and tri-, tetra-, penta-, and hexa-nucleotide motifs with a minimum of five repeats (Zeng et al., 2010; Wei et al., 2011).

### Identification of differentially expressed genes

DESeq was performed to detect the genes which were differentially expressed, based on a threshold false discovery rate (FDR) <0.01 and an absolute value log2ratio ≥2. If the FDR (*Q* = *V*∕*R*) is required to remain below a cutoff (e.g., 0.01), then the FDR can be calculated according to the Benjamini and Hochberg algorithm as: FDR =*E*(*Q*) = *E*{*V*∕(*V* + *S*)} = *E*(*V*∕*R*) (Benjamini & Hochberg, 1995). All of the DEGs were used for the Nr, Swissport, GO, KEGG and COG Functional annotation analyses.

### Sequence retrieval of transcription factors related to NAC, MYB and WRKY

Hidden Markov Model (HMM) was employed factors. The profiles of the NAC, MYB and WRKY DNA-binding domain PF01849, PF00249 and PF03106 used for the HMM search (HMMER 3.1, http://hmmer.janelia.org/) were downloaded from the Pfam database ( http://pfam.sanger.ac.uk/), respectively. There were 5 NAC transcription factors, 190 MYB transcription factors and 34 WRKY transcription factors were obtained with an *E*-value threshold of 0.1. Ultimately, the expression levels of these transcription factors were identified according to the results of DEGs.

### Sequence alignments and phylogenetic constructions of transcription factors

Alignment of the amino acid sequences of the NAC, MYB, WRKY transcription factor domains were aligned with Clustal X using the default parameters. For the phylogenetic analysis, the neighbor-joining trees was constructed by MEGA6.0. Bootstrap values obtained after 1,000 replications are indicated on the branches.

## Results

### RNA-Seq and de novo transcriptome assembly

To obtain a global overview of the Chinese fir transcriptome at different developmental phases, nine RNA samples from immature xylem at three different cultivation stages (fast growing stage, stem growth stage and senescence stage) were sequenced with Illumina HiSeq™ 2500. After stringent quality assessment and data filtering, a total of 68.71 million reads and 13.88 gigabase pairs (Gbp) were generated (Table 1). The reads with base quality greater than 30 (*Q* ≥ 30) and no ambiguous “N” were defined as high-quality reads.

**Table 1:** Summary of Illumina transcriptome sequencing for Chinese fir.

Sample	Total reads	Total bases	GC (%)	Q30%
Chinese fir	68 719 634	13 879 230 822	43.24	96.42

Reads were mapped against the reference transcriptome sequences of Chinese fir. After the removal of adaptor sequences and exclusion of contaminated or short reads, 33,966,473 high-quality reads were assembled into 6,590,556 contigs (https://figshare.com/s/fdf6af8b8ae6aa02bd52) using SOAPdenovo (Li et al., 2010). Using the Trinity *de novo* assembly program, next-generation short-read sequences were assembled into 232,138 transcripts with mean length of 819.51 base pairs (bp) and N50 length of 1,635 bp. The transcripts were subjected to cluster and assembly analyses. Finally 140,486 unigenes with a mean size of 568.64 bp were obtained, these included 17,902 unigenes (12.74%) with length greater than 1 kb. An overview of the contigs, transcripts and unigenes is shown in Table 2.

**Table 2:** Length distribution of assembled contigs, transcripts and unigenes.

Nucleotide length(bp)	Contigs	Transcripts	Unigenes
0–300	6,508,442	87,745	69,385
300–500	42,221	51,541	35,452
500–1,000	21,099	36,109	17,747
1,000–2,000	11,681	31,744	10,593
2,000+	7,113	24,999	7,309
Total number	6,590,556	232,138	140,486
Total length	404,940,125	190,238,773	79,886,333
N50 length	64	1,635	882
Mean length	61.44	819.51	568.64

**Figure 1:**
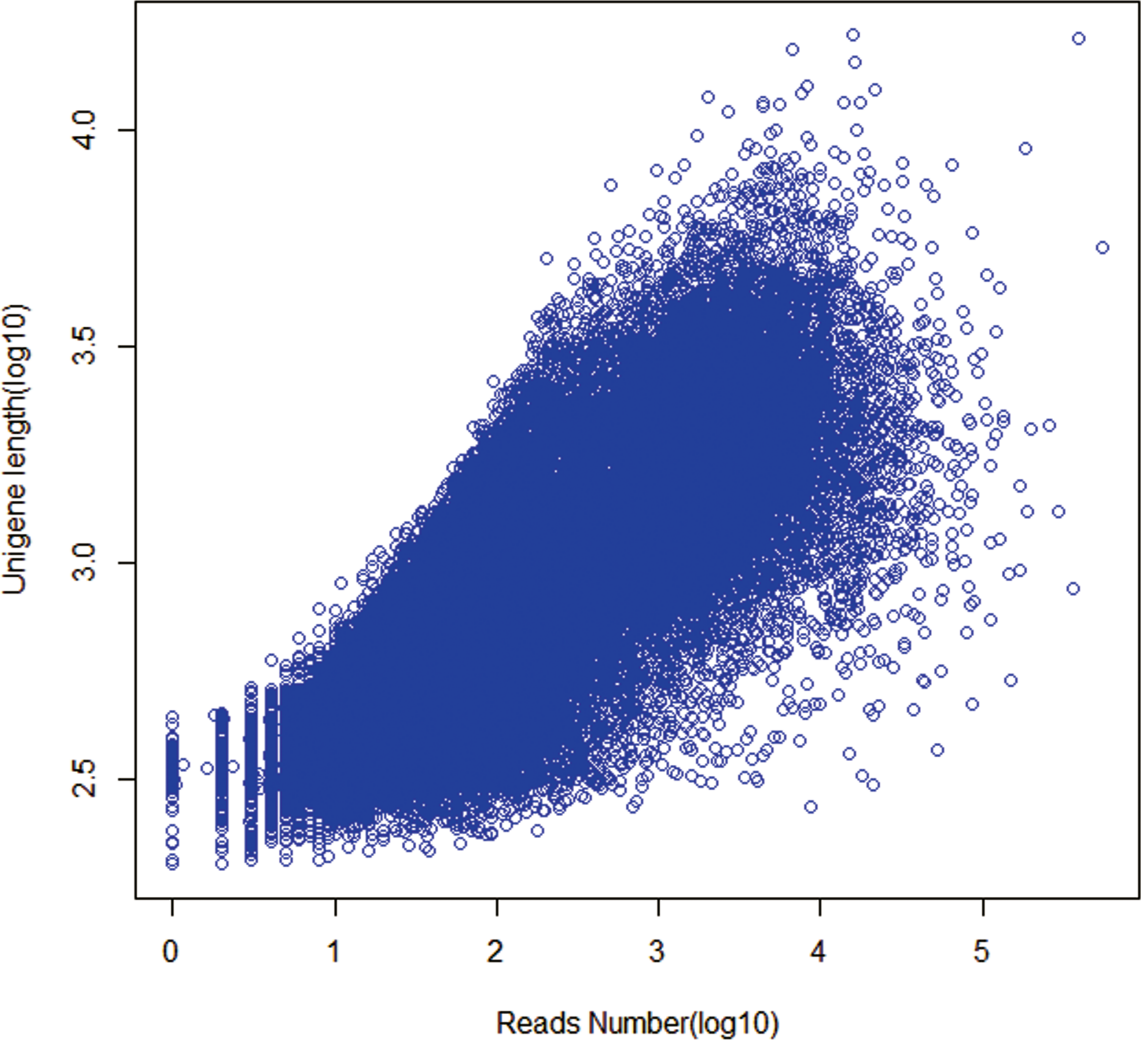
Dependence of unigene lengths on the number of reads assembled into that unigenes.

The length distributions of contigs, transcripts and unigenes were shown in Table 2. As expected for a randomly fragmented transcriptome, there was a positive relationship between the length of a given unigene and the number of reads assembled into it (Fig. 1). Open Reading Frame (ORF) prediction analysis performed with GetORF ( http://emboss. sourceforge.net/apps/cvs/emboss/apps/getorf.html) identified 71,044 unigenes (50.57%) as having ORFs starting with an ‘ATG’ codon. The raw reads of Chinese fir produced in this study have been deposited in the National Center for Biotechnology Information (NCBI) Sequence Read Archive (SRA) database (accession number: SRS959453).

Mapped read depth (reads per kilo base per million reads (RPKM)) was used as a metric for the expression of each unigenes. The expression of the unigenes varied similarly with sequencing depth (Table S1). The expression of unigenes ranged from 0 to 7,443.78 RPKM with an average of 9.32 RPKM. Unigenes with low RPKM values were removed, because they may not have been reliable due to low abundance or statistical errors. Of 71,102 unigenes remaining, 60,631 (85.27%) had a very low expression level of less than 10 RPKM. Unigenes with high RPKM values included those related to metabolism, cell wall biogenesis and remodeling, signal transduction and stress, such as laccase, poly-ubiquitin, ARF-L1 protein, thaumatin-like protein.

### Functional annotation and classification

**Figure 2:**
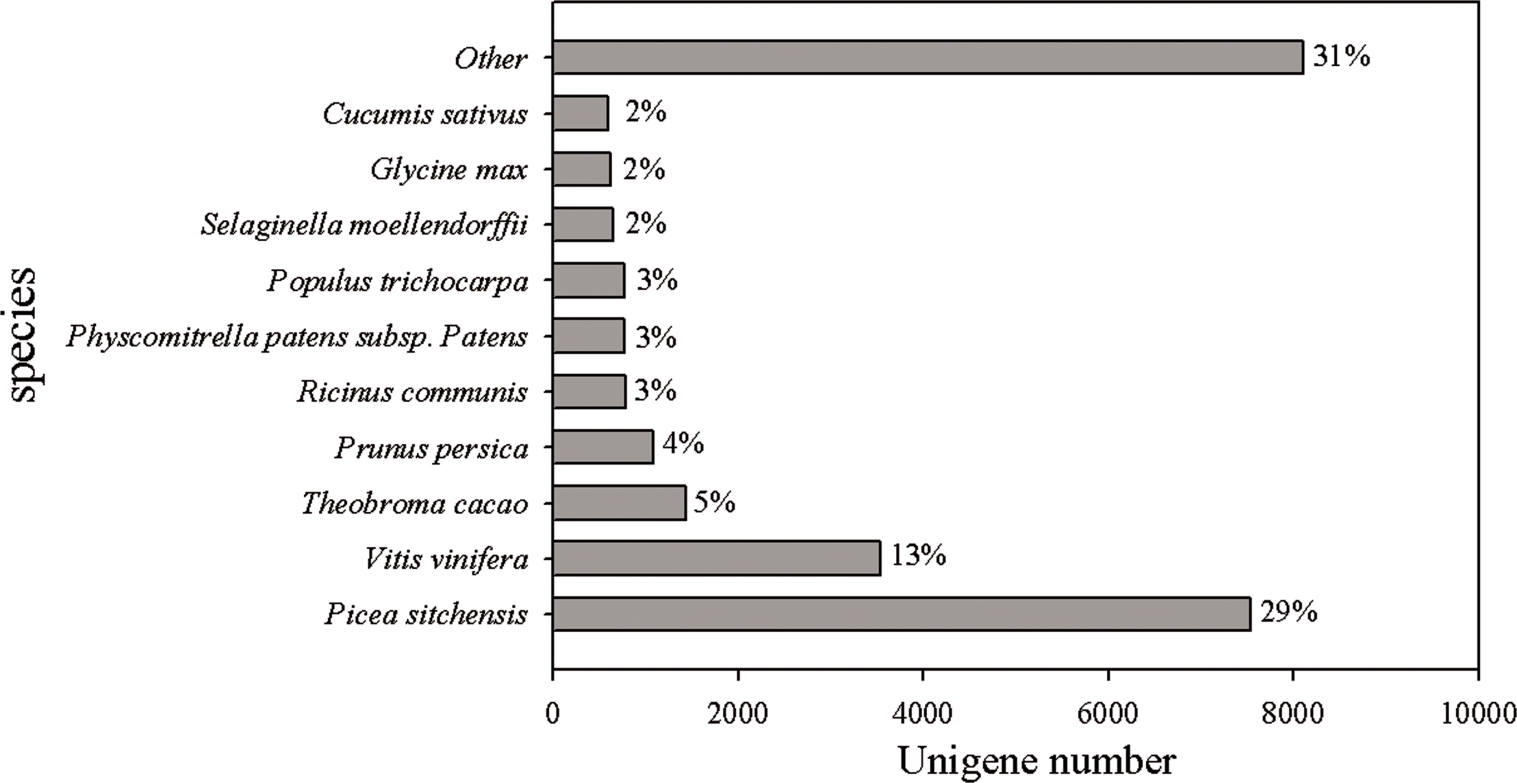
Species distribution of the top BLAST hits in Nr dababase. Top BLASTX results for 26,305 unigenes were calculated. Species with proportions of more than 1% are shown.

The reads of Chinese fir in different cultivation phases (7Y, 15Y and 21Y) were assembled, and several complementary approaches were utilized to annotate the assembled sequences (Table 3). Only 19.52% of the unigenes (27,427) were able to be annotated based on aligning with sequences deposited in diverse protein databases, including the National Center for Biotechnology Information (NCBI) nonredundant protein (Nr) database, Cluster of Orthologous Groups of proteins (COG), Kyoto Encyclopedia of Genes and Genomes (KEGG), and UniProt/Swiss-Prot. According to the BLASTX results, 26,305 (18.72%) unigenes had homologous proteins in the Nr protein database. We found that for 29% of the unigenes the most similar proteins sequence was from *P. sitchensis*, whereas 13% were most similar to sequences from *Vitis vinifera*, and 5% to *Theobroma cacao* (Fig. 2).

**Table 3:** Functional annotation of Chinese fir unigenes.

Annotated databases	Unigenes	≥300 bp	≥1,000 bp	Percentage of unigenes
Nr-annotation	26,305	12,136	14,169	18.72%
COG-annotation	9,942	3,797	6,145	7.08%
GO-annotation	15,085	6,206	8,879	10.74%
KEGG-annotation	5,331	1,854	3,477	3.79%
Swissprot-annotation	17,407	7,276	10,131	12.39%
Total	27,427	12,899	14,528	19.52%

**Figure 3:**
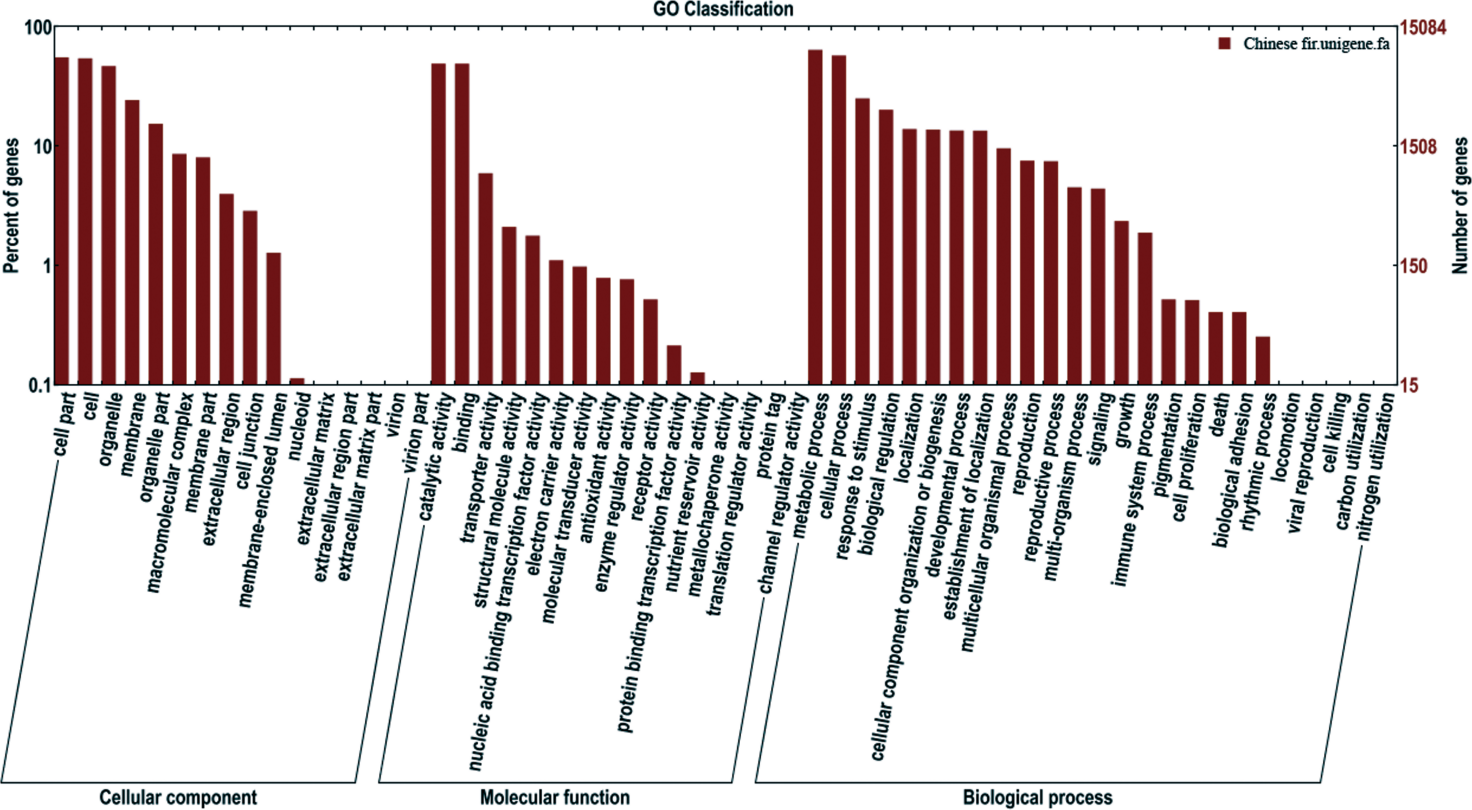
Functional annotation of assembled sequences based on gene ontology (GO) categorization. GO analysis was summarized into three categories: cellular component, molecular function and biological process.

Gene Ontology (GO) analysis was used for functional classification of the assembled transcripts and gene products in terms of their likely associated biological processes, cellular components, and molecular functions. There were 26,305 unigenes annotated in the Nr database, among which 15,085 unigenes were assigned one or more GO terms, with 37.2% in cellular components, 18.9% in molecular functions, and 43.9% in biological processes (Fig. 3). To better review GO cellular components, the GO terms were further clustered to their parent terms. For biological processes, genes involved in metabolic processes, cellular processes, and response to stimulus were highly represented. For molecular functions, catalytic activity, binding, and transporter activity were the most highly represented. For cellular components, the three top classifications were cell part, cell and organelle.

**Figure 4:**
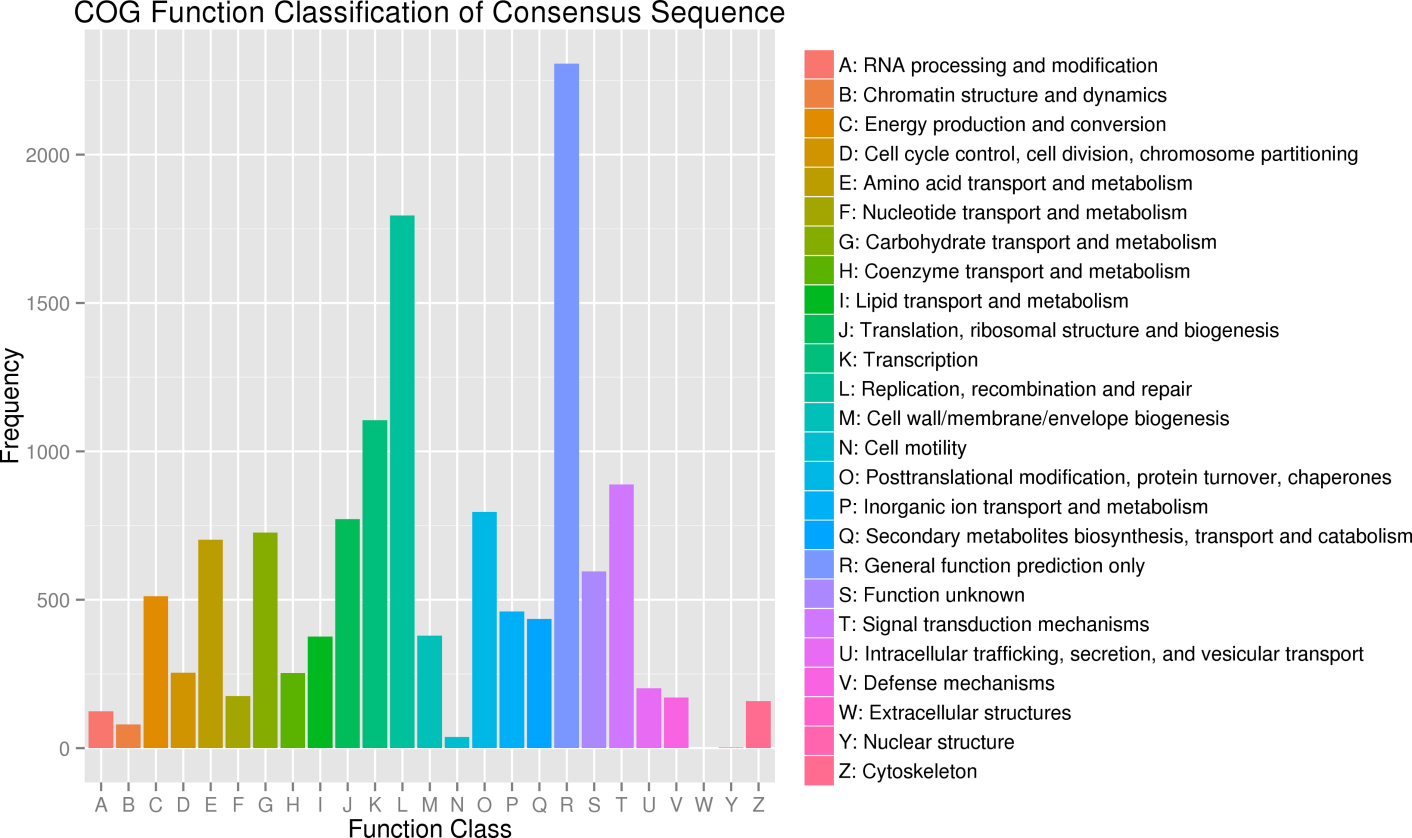
Clusters of orthologous group (COG) classification.

In addition, all unigenes were aligned to the COG database for further functional prediction and classification. Overall, 9,942 of the 140,486 sequences were assigned to 24 COG categories, including RNA processing and modification, chromatin structure and dynamics, energy production and conversion, cell cycle control, cell division, and chromosome partitioning (Fig. 4). The category of general function prediction only represented the largest group (2,306; 17.32%), followed by replication, recombination and repair (1,795; 13.48%), transcription (1,105; 8.30%). Only a few unigenes were assigned to chromatin structure and dynamics, cell motility and nuclear structure (80, 38 and 2 unigenes, respectively). Furthermore, 379 unigenes were assigned to cell wall/membrane/envelope biogenesis and 158 unigenes were assigned to cytoskeleton. No unigene was assigned to extracellular structures.

KEGG is a public database for networks of molecular interactions in cells and their variants specific to particular organisms. To further examine the usefulness of the Chinese fir unigenes generated in the present study, the unigenes were compared with the KEGG database using BLASTX and the corresponding pathways were established. Only 5,331 (3.79%) unigenes were assigned 118 pathways (Table S2). The pathways with highest unigene representation were Ribosome (ko03010, 213 unigenes, 3.69%), followed by RNA transport (ko03013, 195 unigenes, 3.38%) and Spliceosome (ko03040, 173 unigenes, 3.00%).

### SSR marker discovery

SSRs can be used as powerful molecular markers for genetics, evolution and breeding studies. To explore SSR profiles in the unigenes of Chinese fir, the 17,902 unigene sequences were searched for SSRs. In total, 2,784 sequences containing 3,267 SSRs were obtained, with 394 unigene sequences containing more than one SSR. Tri-nucleotide repeat motifs (65.02%) were the most abundant, followed by Di-nucleotide repeats (31.53%) (Table 4). The most abundant repeat type was AAG/CTT (232, 18.24%), followed by AG/CT (166, 13.05%), and AT/AT (161, 12.66%).

**Table 4:** Frequency of candidate SSRs in Chinese fir.

Motif	Repeat number											Total	%
	5	6	7	8	9	10	11	12	13	14	>14		
Di	–	210	73	41	39	15	21	1	0	1	0	401	31.53
Tri	518	196	94	17	1	1	0	0	0	0	0	827	65.02
Tetra	–	23	3	0	1	0	0	0	0	0	0	27	2.12
Penta	–	5	1	0	0	0	0	0	0	0	0	6	0.47
Hexa	–	3	4	3	1	0	0	0	0	0	0	11	0.86
Total	518	437	175	61	42	16	21	1	0	1	0	1,272	100
%	40.72	34.36	13.76	4.80	3.30	1.26	1.65	0.08	0	0.08	0	100	

**Table 5:** Number of up- and down-regulated DEGs in xylem of Chinese fir at different ages.

Comparison	Number of DEGs	Up	Down
7Y vs. 15Y	3	1	2
7Y vs. 21Y	16	14	2
15Y vs. 21Y	5,899	2,623	3,276

**Figure 5:**
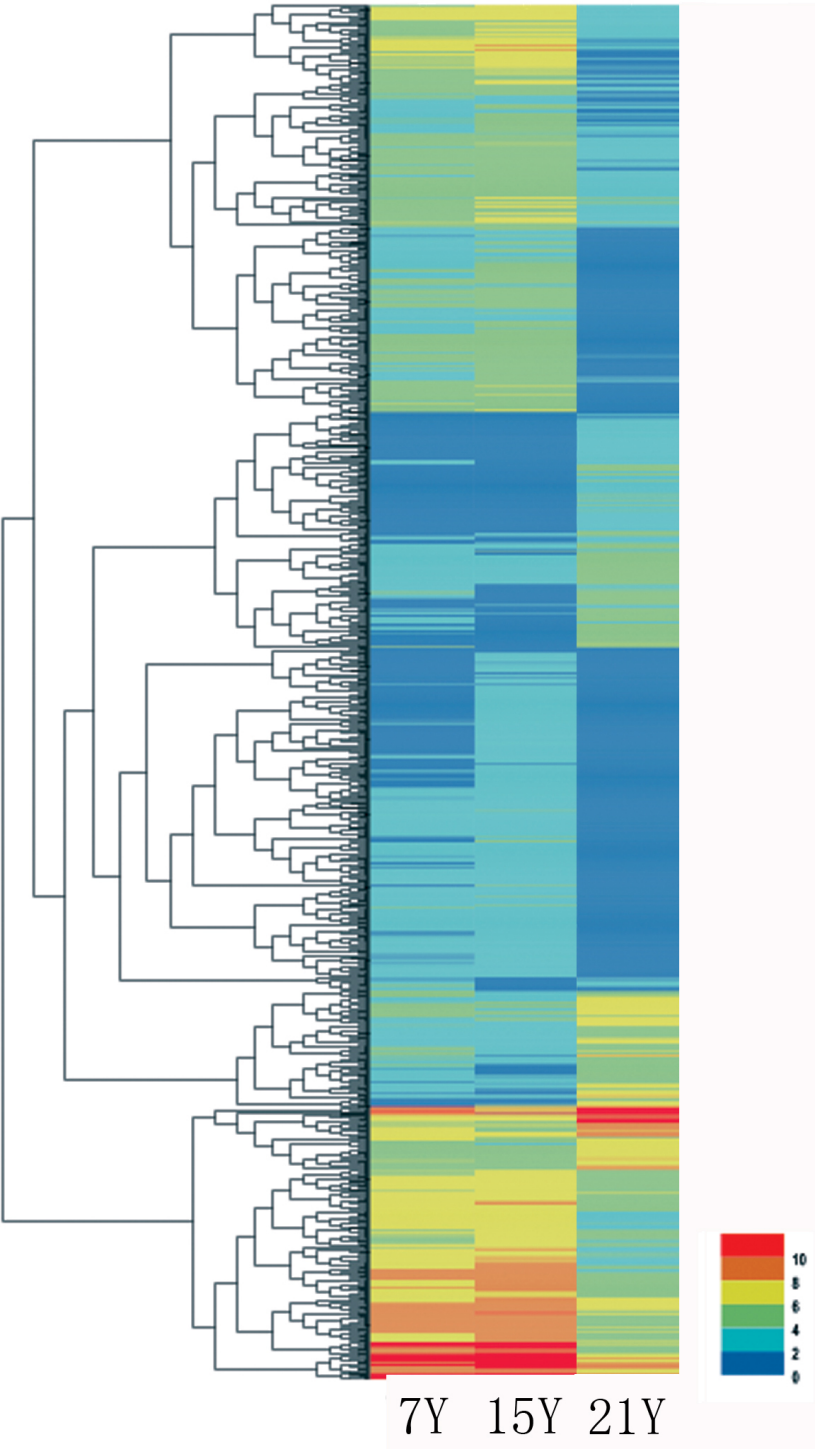
Heatmap of the relative expression levels of differentially expressed genes.

### Identification of differentially expressed genes

A total of 140,486 unigenes were detected from the clean reads of all three samples as described above. To detect DEGs between the samples harvested from tress at different stages, DESeq was used with the criteria FDR ≤ 0.01 and log2Ratio ≥ 2. Whereas only a few DEGs were identified for the comparisons of the samples from 7-year-old trees, by far the most, DEGs were identified from the 15Y vs. 21Y comparisons (Table 5). The representative genes of up- and down-regulated DEGs in xylem of Chinese fir at different phases were shown in Fig. 5 and Table S3. For the 7Y vs. 21Y comparison, most DEGs were up-regulated. Whereas the 15Y vs. 21Y comparison produced a roughly similar number of up-regulated DEGs and down-regulated DEGs.

The up- and down-regulated DEGs were further analyzed based on GO component, GO function and GO process (Tables 6–8). The main GO component categories were cell part, cell, and organelle. In GO function ontology, the major classifications for the DEGs were catalytic activity, binding, and transporter activity. Most of the DEGs were classified into GO process categories of metabolic process, cellular process, and single-organism process. These results indicate that most of the DEGs were related to metabolism, cell wall biogenesis and remodeling, signal transduction and stress.

**Table 6:** Up- and down-regulated Chinese fir DEGs by GO component ontology.

GO component ontology	7Y vs. 15Y			7Y vs. 21Y			15Y vs. 21Y		
	Up	Down	*P*-value	Up	Down	*P*-value	Up	Down	*P*-value
Cell part	0	1	8.72E–01	4	0	5.78E–01	772	747	1.00E+00
Cell	0	1	8.55E–01	3	0	8.97E–01	749	734	1.00E+00
Organelle	0	1	7.36E–01	3	0	7.15E–01	599	591	9.98E–01
Membrane	0	1	3.82E–01	3	0	1.59E–01	345	403	4.27E–09
Organelle part	0	0	1.00E+00	0	0	1.00E+00	234	206	2.02E–01
Macromolecular complex	0	0	1.00E+00	0	0	1.00E+00	117	82	9.91E–01
Membrane part	0	0	1.00E+00	0	0	1.00E+00	107	149	3.44E–04
Cell junction	0	1	4.51E–02	0	0	1.00E+00	60	45	1.70E–04
Extracellular region	0	1	6.24E–02	0	0	1.00E+00	58	98	4.34E–08
Membrane-enclosed lumen	0	0	1.00E+00	0	0	1.00E+00	5	5	1.00E+00
Nucleoid	0	0	1.00E+00	0	0	1.00E+00	3	1	3.49E–01
Extracellular matrix	0	0	1.00E+00	0	0	1.00E+00	1	3	2.22E–01
Extracellular matrix part	0	0	1.00E+00	0	0	1.00E+00	0	1	6.20E–01
Extracellular region part	0	0	1.00E+00	0	0	1.00E+00	0	8	9.85E–04

**Table 7:** Up- and down-regulated Chinese fir DEGs by GO function ontology.

GO function ontology	7Y vs. 15Y		7Y vs. 21Y		15Y vs. 21Y	
	Up	Down	Up	Down	Up	Down
Catalytic activity	0	1	0	1	600	657
Binding	0	1	1	0	511	561
Transporter activity	0	0	1	0	76	86
Structural molecule activity	0	0	0	0	56	20
Nucleic acid binding transcription factor activity	0	0	0	0	32	29
Electron carrier activity	0	0	0	0	21	15
Molecular transducer activity	0	0	0	0	14	15
Enzyme regulator activity	0	0	0	0	11	8
Antioxidant activity	0	0	0	0	10	9
Receptor activity	0	0	0	0	4	4
Guanyl-nucleotide exchange factor activity	0	0	0	0	2	2
Protein binding transcription factor activity	0	0	0	0	1	0
Nutrient reservoir activity	0	0	0	0	1	1
Metallochaperone activity	0	0	0	0	0	1

**Table 8:** Up- and down-regulated Chinese fir DEGs by GO process ontology.

GO function ontology	7Y vs. 15Y		7Y vs. 21Y		15Y vs. 21Y	
	Up	Down	Up	Down	Up	Down
Metabolic process	0	1	2	1	782	765
Cellular process	0	1	2	0	661	718
Single-organism process	0	1	1	0	601	672
Response to stimulus	0	1	0	0	357	354
Biological regulation	0	1	0	0	281	278
Localization	0	1	1	0	194	222
Cellular component organization or biogenesis	0	1	0	0	177	188
Developmental process	0	1	0	0	172	211
Multicellular organismal process	0	1	0	0	132	131
Reproductive process	0	1	0	0	89	102
Multi-organism process	0	1	0	0	59	75
Signaling	0	0	0	0	53	75
Growth	0	1	0	0	36	62
Reproduction	0	0	0	0	28	27
Immune system process	0	0	0	0	23	28
Biological adhesion	0	0	0	0	6	15
Rhythmic process	0	0	0	0	3	5
Biological phase	0	0	0	0	2	1
Locomotion	0	0	0	0	1	2

### Transcription factors of interest

**Figure 6:**
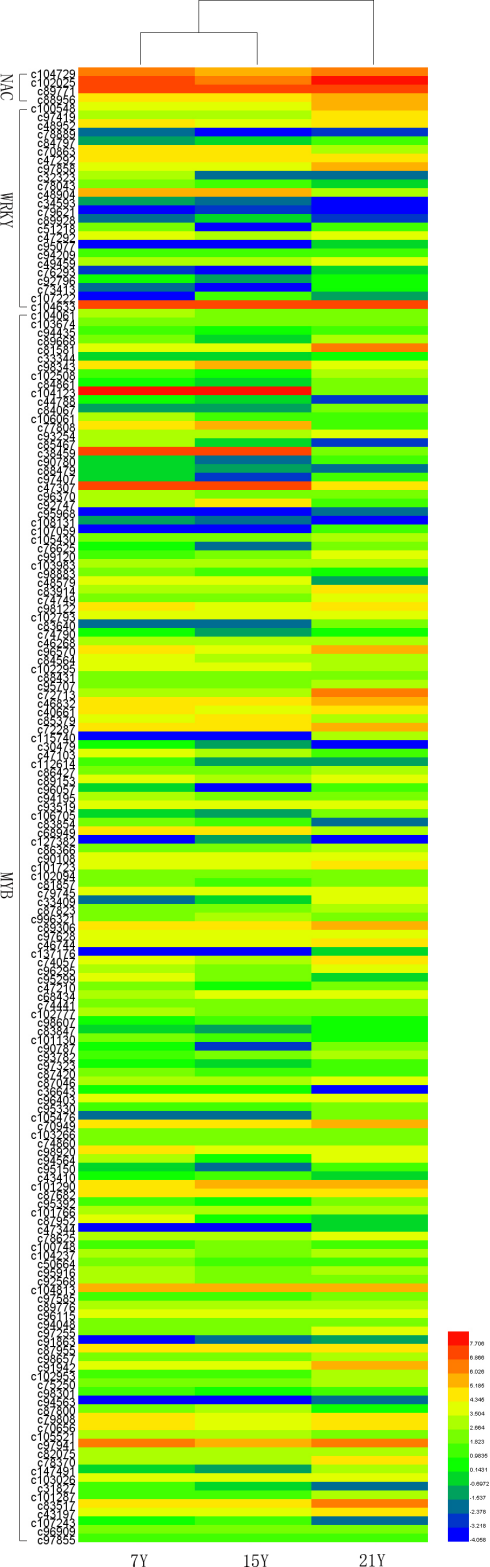
The differential expression patterns of representative TFs in different developmental phases.

Recent studies have demonstrated that many transcription factors, such as NAC, MYB, and WRKY gene families, regulate the formation of secondary wall (Rogers & Campbell, 2004; Kubo et al., 2005; Zhong & Ye, 2009; Wang et al., 2010a). Five NAC transcription factors, 190 MYB transcription factors, and 34 WRKY transcription factors were identified respectively from Chinese fir transcriptome. Phylogenetic analyses of the three clusters of transcription factors were shown in the Figs. S1–S3. The transcription factors of NAC, MYB, WRKY were clustered into two, three, three main classes separately. The expression profiles of differentially expressed transcription factors were analyzed in different developmental phases (Fig. 6). The transcript levels of 32 MYB and 3 WRKY transcription factors increased from 7Y to 21Y, while 28 MYB and 4 WRKY exhibited a reduced expression profiles. We chose all of the NAC, WRKY transcription factors and 60 MYB transcription factors (full sequence score >100) matching to the DEGs results, and discovered five WRKY transcription factors including three up-regulated DEGs (c100548.graph_c0, c49459.graph_c0, c97419.graph_c0) and two down-regulated DEGs (c48904.graph_c0, c70863.graph_c0). Furthermore, 17 MYB transcription factors were dug out including five up-regulated DEGs (c89668.graph_c0, c84067.graph_c0, c90780.graph_c0, etc.) and 12 down-regulated DEGs (c104123.graph_c0, c106541.graph_c0, c98343.graph_c0, etc.) in 15Y vs. 21Y (Table S4).

## Discussion

RNA-seq has emerged to be a valuable tool to discover molecular markers and identify novel genes. In recent years, the growing number of species for which significant genetic resources are available is sparking a new era of plant genetic study (Morozova & Marra, 2008; Coppe et al., 2010). In this study, RNA-seq technology was applied to the Chinese fir transcriptome using Illumina HiSeq™ 2500 platform, and the transcriptome at three different cultivation phases was systematically investigated.

A total of 68.71 million reads and 13.88 gigabase pairs (Gbp) were generated, and 140,486 unigenes with a mean size of 568.64 bp were obtained. About 19.52% of the unigenes were successfully annotated, and the information regarding putative nucleotide variations offers intriguing leads for the analysis of the transcriptome and biological functions in Chinese fir. Among the unigenes 29%, 13%, and 5% appeared to be most closely related to genes from *P. sitchensis, Vitis vinifera*, and *Theobroma cacao* respectively. Chinese fir and *P. sitchensis* gymnosperms assigned to the Coniferopsida Coniferae. Thus, there is a close relationship between *P. sitchensis* and Chinese fir based on both systematic botany and molecular analysis. This research moves us toward identifying candidate genes for wood formation and clarifying the functions of the relevant pathways in Chinese fir. Compared with those from other conifer trees, our results using samples from different cultivation phases identified a much larger number of unigenes. In addition, the mean length of unigenes (568 bp) that we obtained is much longer than those in previous studies using the same technology, which reported 449 bp (Huang et al., 2012), 505 bp (Wang et al., 2013a), and 497 bp (Qiu et al., 2013). To the best of our knowledge, this study represents the first attempt at de novo sequencing and assembly of the Chinese fir trancriptome using RNA-seq, to focus on different cultivation phases including fast growing stage, stem growth stage and senescence stage respectively. The results obtained in this research demonstrated that our final assembly quality was satisfactory and it therefore provides sequence resources and facilitates further gene cloning and functional analyses.

Several genes encoding the biosynthesis of wood components (cellulose, xylan, glucomannan, and lignin), such as Cel/TDIF/CLE/PXY-WOX4/MYB (Plomion, Leprovost & Stokes, 2001; Ito et al., 2006; Hirakawa et al., 2008; Etchells & Turner, 2010; Hirakawa, Kondo & Fukuda, 2010; Ji et al., 2010; Suer et al., 2011; Wang et al., 2011), have been identified in angiosperms (Jansson & Douglas, 2007). Unfortunately, only few of the related genes have been identified and functionally characterized in gymnosperms. In this study, the lack of a reference genome for Chinese fir, hampered our efforts to determine gene and their functions. BLAST hits Perhaps these unigenes might play an important roles in Chinese fir and quite different from the other species.

Differences in gene expression profiles can yield insight into mechanisms underlying physiological changes, and DEGs were found among different developmental stages, tissues, treatments, and species (Zeng et al., 2010; Logacheva et al., 2011; Wang et al., 2013b; Wu et al., 2013; Wu et al., 2014). The 7Y, 15Y and 21Y samples that we collected represented the different cultivation development phases (fast growing stage, stem growth stage and senescence stage), and there were vary a few DEGs in 7Y compared to either 15Y or 21Y. However, many up-regulated (2,623) and down-regulated (3,276) DEGs were detected in 15Y compared to 21Y. Furthermore, most DEG unigenes in 15Y vs. 21Y were annotated to specific pathways using the KEGG database, including metabolic pathways, and biosynthesis of secondary metabolites among others. These results indicate considerable changes of gene expression in immature xylem during the transition from stem growth stage and senescence stage. A similar phenomenon was reported for the transition from the active stage to the dormant stage in vascular cambium, in which expression of the core cell cycle genes in vascular cambium correlated well with the cessation of cambial cell division (Brown et al., 2005; Druart et al., 2007; Li et al., 2009; Galindo González et al., 2012; Qiu et al., 2013). We can conclude that the key cycle protein transcripts expressed preferentially at stem growth stage and senescence stage of Chinese fir may be essential for wood formation, and these DEGs are involved in a broad range of physiological functions between stem growth stage and senescence stage.

##  Supplemental Information

10.7717/peerj.2097/supp-1 Table S1 Assessment of assembly quality

10.7717/peerj.2097/supp-2 Table S2 Pathway assignment based on KEGG

10.7717/peerj.2097/supp-3 Table S3 The representative genes of up- and down-regulated DEGs in xylem of the Chinese fir at different developmental phases

10.7717/peerj.2097/supp-4 Table S4 MYB, WRKY transcriptome factors in DEGs

10.7717/peerj.2097/supp-5 Figure S1 Phylogenetic tree of NAC transcription factors

10.7717/peerj.2097/supp-6 Figure S2 Phylogenetic tree of MYB transcription factors

10.7717/peerj.2097/supp-7 Figure S3 Phylogenetic tree of WRKY transcription factors

## Additional Information and Declarations

### Data Availability

The following information was supplied regarding data availability: The National Center for Biotechnology Information (NCBI) Sequence Read Archive (SRA) database (accession number: SRS959453). Assembly contigs can be found in Figshare: https://figshare.com/s/fdf6af8b8ae6aa02bd52.
