# Supplementary material for: Transcriptome analysis of immature xylem in the Chinese fir at different developmental phases
Source: PeerJ. 2016 Jun 7;4:e2097. doi: 10.7717/peerj.2097 (PMC4906661; doi:10.7717/peerj.2097)
Supplement: Table S3 [file peerj-04-2097-s003.docx]

**Table S4** **the representative genes of up- and down-regulated DEGs in xylem of Chinese fir at different phases**

| 7Y vs. 15Y | | 7Y vs. 21Y | | 7Y vs. 21Y | |
| --- | --- | --- | --- | --- | --- |
| Up-regulated genes | Down-regulated genes | Up-regulated genes | Down-regulated genes | Up-regulated genes | Down-regulated genes |
| c102369.graph_c0 | c98589.graph_c0 c92682.graph_c0 | c104277.graph_c0 c41392.graph_c0 c42066.graph_c0 c47049.graph_c0 c57402.graph_c0 c65951.graph_c0 c73769.graph_c0 c78276.graph_c0 c84116.graph_c0 c85267.graph_c0 c87436.graph_c0 c90898.graph_c0 c98674.graph_c0 c98823.graph_c0 | c100636.graph_c0  c103311.graph_c0 | c100000.graph_c0 c100000.graph_c1 c100033.graph_c0 c100040.graph_c0 c100043.graph_c0 c100047.graph_c0 c100077.graph_c0 c100082.graph_c0 c100086.graph_c0 c100088.graph_c0 c100095.graph_c0 c100109.graph_c0 c100128.graph_c0 c100130.graph_c0 c100358.graph_c0 c100361.graph_c0 c100391.graph_c0 | c100008.graph_c0 c100048.graph_c0 c100052.graph_c1 c100068.graph_c0 c100104.graph_c0 c100136.graph_c0 c100145.graph_c0 c100154.graph_c0 c100167.graph_c0 c100184.graph_c0 c100200.graph_c1 c100213.graph_c0 c100225.graph_c0 c100227.graph_c0 c100243.graph_c0 c100246.graph_c0 c100260.graph_c0 |
