# Supplementary figures and images for: Transcriptome analysis of immature xylem in the Chinese fir at different developmental phases
Source: PeerJ. 2016 Jun 7;4:e2097. doi: 10.7717/peerj.2097 (PMC4906661; doi:10.7717/peerj.2097)

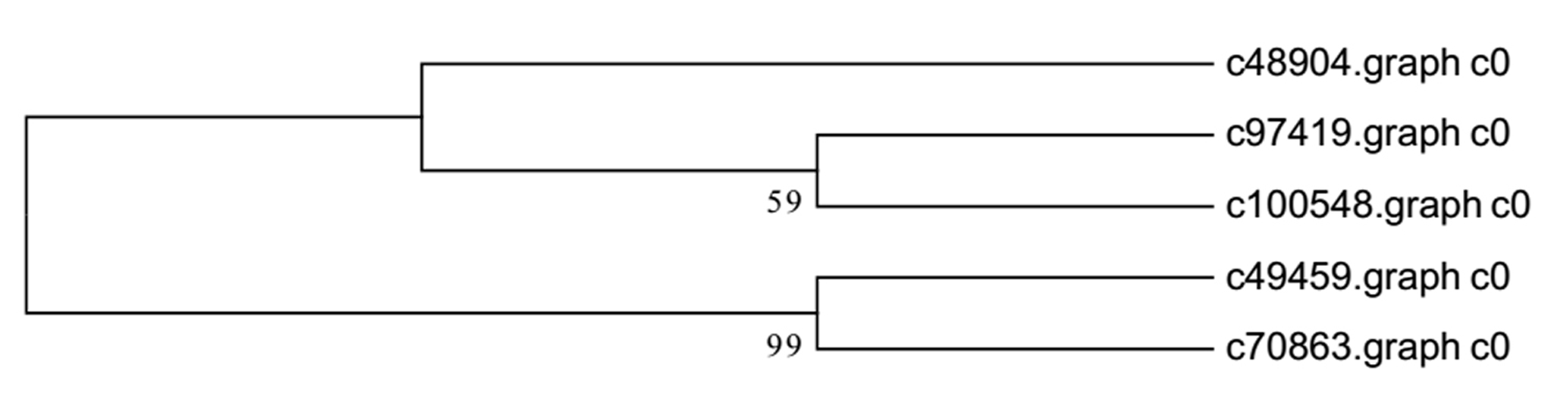

Supplement: Figure S1 [file peerj-04-2097-s005.png]

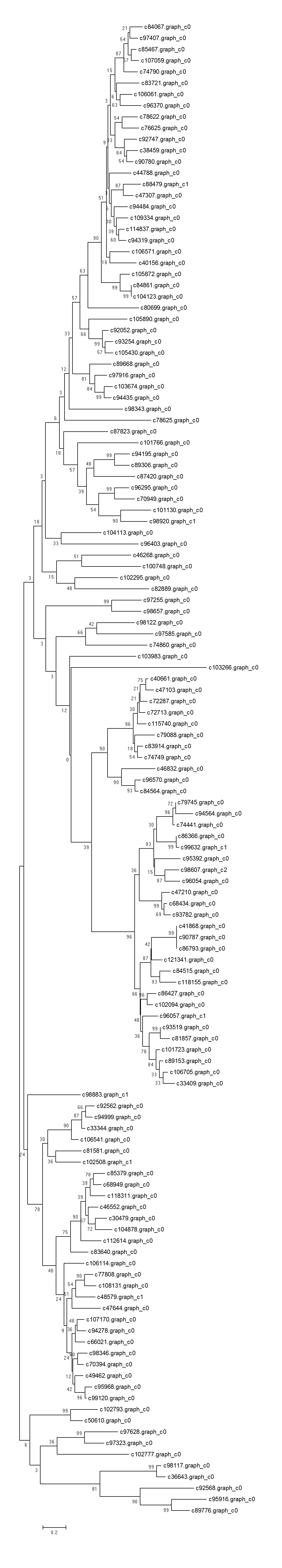

Supplement: Figure S2 [file peerj-04-2097-s006.png]

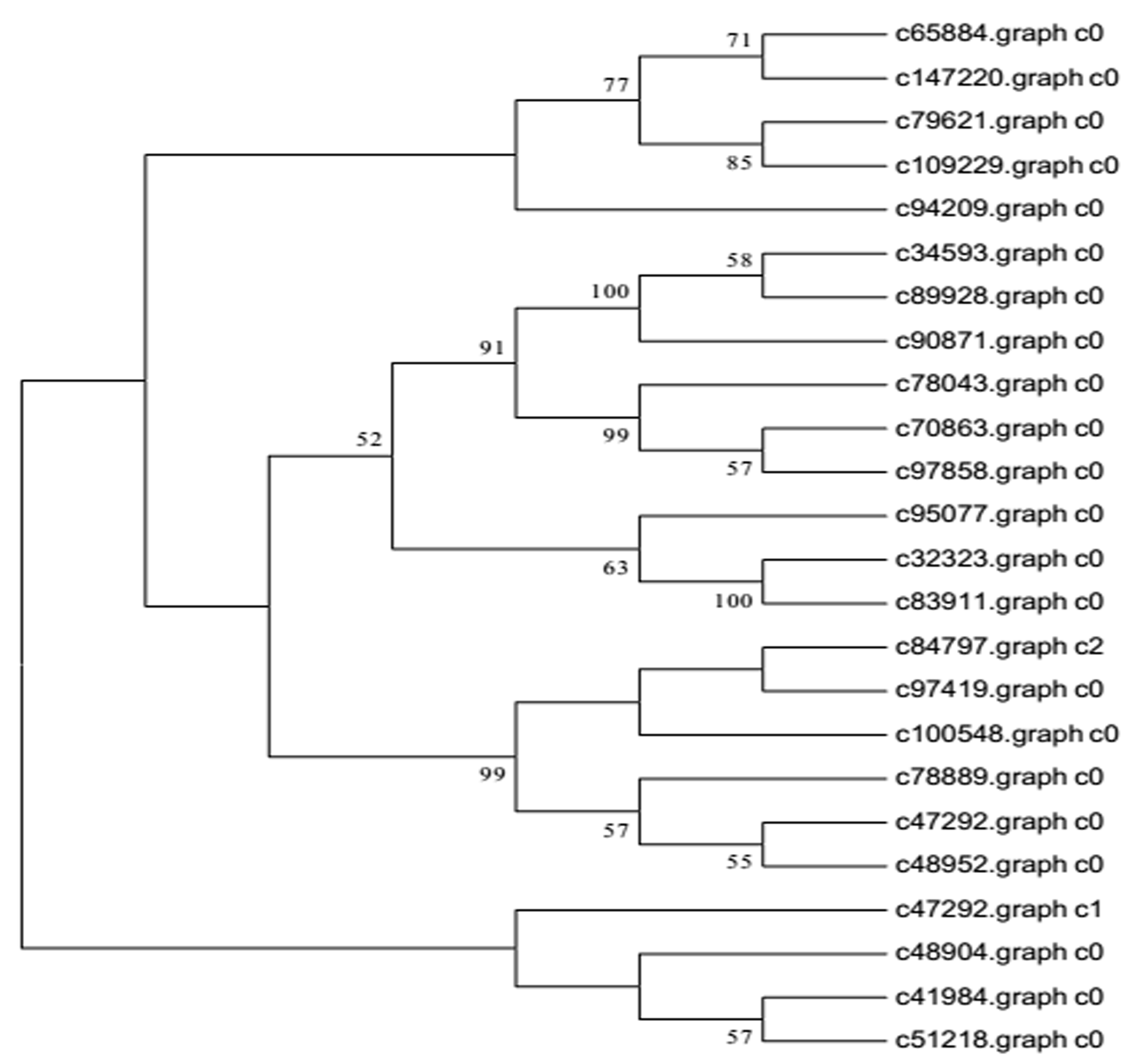

Supplement: Figure S3 [file peerj-04-2097-s007.png]
